# Evaluation of Etest and MICRONAUT-AM Assay for Antifungal Susceptibility Testing of *Candida auris*: Underestimation of Fluconazole Resistance by MICRONAUT-AM and Overestimation of Amphotericin B Resistance by Etest

**DOI:** 10.3390/antibiotics13090840

**Published:** 2024-09-04

**Authors:** Mohammad Asadzadeh, Suhail Ahmad, Wadha Alfouzan, Inaam Al-Obaid, Bram Spruijtenburg, Eelco F. J. Meijer, Jacques F. Meis, Eiman Mokaddas

**Affiliations:** 1 Department of Microbiology, Faculty of Medicine, Kuwait University, Safat 13110, Kuwait; mohammad.assadzadeh@ku.edu.kw (M.A.); alfouzan.w@ku.edu.kw (W.A.); eiman.mokaddas@ku.edu.kw (E.M.); 2 Microbiology Department, Farwaniya Hospital, Farwaniya 81004, Kuwait; 3 Microbiology Department, Al-Sabah Hospital, Shuwaikh 70031, Kuwait; inaob218@hotmail.com; 4 Canisius Wilhelmina Hospital (CWZ)/Dicoon, 6532 Nijmegen, The Netherlandseelco.meijer@cwz.nl (E.F.J.M.); 5 Radboudumc-CWZ Center of Expertise for Mycology, 6500 Nijmegen, The Netherlands; jacques.meis@gmail.com; 6 Institute of Translational Research, Cologne Excellence Cluster on Cellular Stress Responses in Aging-Associated Diseases (CECAD) and Excellence Center for Medical Mycology, University of Cologne, 50923 Cologne, Germany; 7 Microbiology Department, Ibn-Sina Hospital, Shuwaikh 70031, Kuwait

**Keywords:** *Candida auris*, antifungal susceptibility testing, Etest, MICRONAUT-AM EUCAST assay, comparative performance

## Abstract

Multidrug-resistant *Candida auris* has recently caused major outbreaks in healthcare facilities. Rapid and accurate antifungal susceptibility testing (AST) of *C. auris* is crucial for proper management of invasive infections. The Commercial Sensititre Yeast One and Vitek 2 methods underestimate or overestimate the resistance of *C. auris* to fluconazole and amphotericin B (AMB). This study evaluated the AST results of *C. auris* against fluconazole and AMB by gradient-MIC-strip (Etest) and broth microdilution-based MICRONAUT-AM-EUCAST (MCN-AM) assays. Clinical *C. auris* isolates (*n* = 121) identified by phenotypic and molecular methods were tested. Essential agreement (EA, ±1 two-fold dilution) between the two methods and categorical agreement (CA) based on the Centers for Disease Control and Prevention’s (CDC’s) tentative resistance breakpoints were determined. Fluconazole resistance-associated mutations were detected by PCR-sequencing of *ERG11*. All isolates identified as *C. auris* belonged to South Asian clade I and contained the *ERG11* Y132F or K143R mutation. The Etest–MCN-AM EA was poor (33%) for fluconazole and moderate (76%) for AMB. The CA for fluconazole was higher (94.2%, 7 discrepancies) than for AMB (91.7%, 10 discrepancies). Discrepancies were reduced when an MCN-AM upper-limit value of 4 µg/mL for fluconazole-susceptible *C. auris* and an Etest upper-limit value of 8 µg/mL for the wild type for AMB were used. Our data show that resistance to fluconazole was underestimated by MCN-AM, while resistance to AMB was overestimated by Etest when using the CDC’s tentative resistance breakpoints of ≥32 µg/mL for fluconazole and ≥2 µg/mL for AMB. Method-specific resistance breakpoints should be devised for accurate AST of clinical *C. auris* isolates for proper patient management.

## 1. Introduction

*Candida auris* is a recently emerged multidrug-resistant yeast pathogen that has caused major outbreaks associated with high mortality rates in healthcare facilities in many countries [1,2,3,4,5]. Although described as a novel species only in 2009 [6], this notorious yeast pathogen has spread globally and has recently been declared as an urgent antimicrobial resistance threat by the Centers for Disease Control and Prevention (CDC) and placed in the critical group of the fungal priority pathogen list by the World Health Organization (WHO) [3,4,7,8]. *C. auris* easily colonizes and sheds from human skin, has demonstrated prolonged survival in the environment, and is also resistant to killing by commonly used disinfectants [9,10]. These features promote persistence and easy transmission of the yeast to other hospitalized patients, causing outbreaks which have been difficult to control [3,5,9,10,11,12,13,14,15]. Whole genome sequencing-based studies have identified six genetically distinct clades, and the vast majority of clinical *C. auris* isolates from South Asia and the Middle East exhibit resistance to fluconazole and belong to clade I [16,17,18,19,20,21]. Therapeutic options for invasive *C. auris* infections are limited as many isolates exhibit resistance to one or more drugs, and there are only four classes of antifungal drugs [2,8,22]. Echinocandins are the first-line treatment for invasive *C. auris* infections; however, resistance can develop during treatment [17,19,22,23]. Rapid diagnosis and accurate antifungal susceptibility testing (AST) of *C. auris* isolates to commonly used antifungal drugs are crucial for proper management and improved outcomes of invasive infections [24,25]. 

Although reference broth microdilution-based protocols of the Clinical Laboratory Standard Institute (CLSI) and the European Committee for Antimicrobial Susceptibility Testing (EUCAST) are the methods of choice for accurate AST of yeast isolates, they are time-consuming and labor-intensive and are not readily available in many routine mycology laboratories. To overcome these difficulties, several commercial methods, such as the Sensititre YeastOne (SYO), Etest and Vitek 2 system (Vitek 2), have been developed for obtaining rapid AST results [26,27,28,29,30]. However, recent studies have shown that Vitek 2 underestimates fluconazole resistance, while both Vitek 2 and colorimetric use of SYO overestimate amphotericin B (AMB) resistance among clinical *C. auris* isolates [31,32]. On the other hand, excellent agreement has been reported within ±2 two-fold dilutions for echinocandins (caspofungin and micafungin) between the reference CLSI method and the commercial Vitek 2 method. Furthermore, the minimum inhibitory concentration (MIC) values of both caspofungin and micafungin did not vary significantly between the two AST methods, and the categorical agreement (CA) between the two methods was almost perfect (99%) with no major errors [32]. Employing the EUCAST methodology, the colorimetric broth microdilution-based MICRONAUT-AM (MCN-AM) assay is another commercial test for determining the MIC values of yeasts to antifungal drugs. This study evaluated the AST results of clinical *C. auris* isolates against fluconazole and AMB by Etest and MCN-AM tests and correlated the AST data with alterations in *ERG11* for fluconazole and mutations in the *ERG6* gene for AMB.

## 2. Results

### 2.1. Species-Specific Identification of C. auris Isolates

All 121 isolates used in this study were identified as *C. auris* by a Vitek 2 yeast identification system, MALDI-TOF MS, and PCR amplification of the ITS region of rDNA. In addition, the DNA sequence data of the ITS region of rDNA matched perfectly (100% identity) with the corresponding *C. auris* sequences described previously from Kuwait (represented by *C. auris* isolate Kw1732/14; GenBank accession no. LN624638) [33,34,35] and India [36] as well as several other (CBS12874, CBS12875, CBS12876, CBS12880, CBS12882, CBS12886 and CBS12887) *C. auris* strains. Furthermore, all 121 isolates used in this study belonged to the South Asian clade I as determined by the 12-loci STR genotyping, as illustrated previously [17,19].

### 2.2. AST for Fluconazole and AMB by Etest and MCN-AM Methods

The MIC values of 121 *C. auris* isolates for fluconazole and AMB by the Etest and MCN-AM assay are presented in Table 1, while the MIC range, modal MIC, MIC_50_, MIC_90_ and the geometric mean values are shown in Table 2. Using the CDC’s tentative resistance breakpoint of ≥32 µg/mL for fluconazole, 119 *C. auris* isolates were resistant to fluconazole, while 2 isolates were classified as fluconazole-susceptible by Etest. On the contrary, 112 isolates were fluconazole-resistant, while 9 isolates were fluconazole-susceptible according to the MCN-AM assay (Table 1). The MIC range, modal MIC, MIC_50_, MIC_90_ and the geometric mean values for fluconazole were lower for the MCN-AM assay compared to Etest (Table 2). The CA for fluconazole between the Etest and MCN-AM assay was 94.2%; however, the essential agreement (EA, ±1 two-fold dilution) between the Etest and MCN-AM assay was poor (30 of 121, 33.1%). Similarly, the Kappa coefficient (κ) value of only 0.159 (95% CI −0.142 to 0.46) also indicated only a slight agreement between the two methods. DNA sequencing of the hotspot region of *ERG11*, involved in conferring resistance to fluconazole [33,36,37], indicated the presence of the Y132F mutation in 110 isolates and K143R mutation in 11 isolates. 

Using the CDC’s tentative resistance breakpoint of ≥2 µg/mL for AMB, 108 *C. auris* isolates were detected as AMB-susceptible, while 13 isolates were scored as AMB-resistant by Etest. On the contrary, 119 isolates were AMB-susceptible, while only 3 isolates were AMB-resistant by the MCN-AM assay (Table 1). All three isolates scored as AMB-resistant by the MCN-AM assay were also AMB-resistant by Etest. The modal MIC, MIC_90_ and the geometric mean values for AMB were lower, while MIC_50_ was higher for the MCN-AM assay compared to Etest (Table 2). The CA for AMB between the Etest and MCN-AM assay was 91.7%, while EA was moderate (84 of 121, 76%). The Kappa coefficient (κ) value of 0.349 (95% CI 0.055 to 0.642) indicated fair agreement between the two methods. DNA sequencing of *ERG6*, involved in conferring resistance to AMB in some yeasts, in all three isolates scored as AMB-resistant by both methods indicated the presence of a deletion frame shift mutation which abrogated its function, as was also determined recently by whole genome sequencing [38].

The CA for fluconazole was higher at 94.2%, with 7 discrepancies, compared to 91.7% for AMB, which had 10 discrepancies. However, the number of discrepancies for fluconazole were reduced to only two (CA of 98.3%) when the MCN-AM upper-limit value of 8 µg/mL or 16 µg/mL was used for fluconazole-susceptible *C. auris*. Similarly, the CA for AMB improved to 100% with no discrepancies when the Etest upper-limit value of 8 µg/mL was used for AMB-susceptible *C. auris*.

## 3. Discussion

Clinical *C. auris* isolates usually exhibit resistance to one or more antifungal drugs [9,22]. Rapid diagnosis and accurate AST of clinical *C. auris* isolates to commonly used antifungal drugs is, therefore, crucial for proper management and improved outcomes of invasive infections caused by this notorious yeast [24,25]. Although reference CLSI and EUCAST are the methods of choice for accurate AST of clinical yeast isolates for proper patient management, they are not usually used by routine mycology laboratories due to their complicated, labor-intensive and time-consuming protocols. Instead, easy-to-use commercial methods such as Etest, Vitek 2 and SYO are preferred for the rapid acquisition of in vitro susceptibility testing data to guide therapy [26,27,28,29,30]. However, recent studies have shown that Vitek 2 underestimates fluconazole resistance, while both Vitek 2 and SYO overestimate AMB resistance of clinical *C. auris* isolates [31,32]. Based on EUCAST methodology, the MCN-AM assay is another recently developed commercial test for determining the MIC values of yeast species isolates to antifungal drugs. To overcome the problems associated with rapid and accurate AST of clinical *C. auris* isolates against fluconazole and AMB by SYO and Vitek 2, we performed a comparative evaluation of two other commercial tests, the Etest and MCN-AM assay, for proper patient management. All 121 isolates used in this study were identified as *C. auris* by a combination of phenotypic and molecular methods and belonged to the South Asian clade I by 12-loci-based STR typing [16,17,19,39]. 

The fluconazole MIC values obtained by Etest were generally higher than the MCN-AM fluconazole MICs with only 33.1% EA (±1 two-fold dilution), which also resulted in a Kappa coefficient (κ) value of only 0.159 (only slight agreement). However, with seven discrepant results, the CA between the two tests was 94.2%. It is pertinent to mention here that CA is a measure of categorical interpretation of the MIC values (i.e., susceptible, intermediate/susceptible (dose-dependent) or resistant) and the CA of the test method relative to the reference method should ideally be ≥90%. Similarly, the test method should also yield EA (i.e., MIC value within ±1 two-fold dilution of the value obtained with the reference method) of ≥90% [40]. Fluconazole-resistant *C. auris* isolates contain the Y132F or K143R mutation in the *ERG11* gene, while fluconazole-susceptible isolates lack these mutations [36,37,41]. However, *ERG11* mutations alone usually confer low-level resistance to fluconazole. High-level fluconazole resistance in *C. auris* usually also involves mutations in other genes such as *TAC1B* or *MRR1A* which upregulate efflux pump genes or due to increase in *ERG11* copy number [22]. Considering that all 121 isolates used in this study belonged to the South Asian clade I, which is usually resistant to fluconazole [16,17,19,24,36], and DNA sequencing data showed the presence of the fluconazole resistance-conferring Y132F or K143R mutation in *ERG11* [37,41] in all the isolates, the interpretation errors (discrepancies) are likely due to an underestimation of fluconazole resistance by the MCN-AM assay. The number of discrepancies was reduced when the MCN-AM-specific upper-limit value of ≤8 µg/mL was used to define fluconazole-susceptible *C. auris*. Two recent studies have also shown that Vitek 2 underestimates fluconazole resistance in *C. auris* [29,32]. Using *C. auris* isolates belonging to five different clades, Siopi et al. [32] have recently shown that automated Vitek 2 also underestimates fluconazole resistance in *C. auris*. The Vitek 2-derived MIC values for fluconazole were considerably lower than the reference CLSI method, and the number of discrepancies was reduced (i.e., CA increased) when a lower MIC value (4 µg/mL) was used to define wild-type strains [32]. 

The AMB MIC values derived from the Etest were also generally higher than the MCN-AM AMB MICs; however, there was considerably higher (76%) EA between the two tests, and the Kappa coefficient (κ) value of 0.349 also indicated fair agreement between the two methods. Despite this, the CA for AMB between the two tests was 91.7% (10 discrepancies). The discrepancies were eliminated when an Etest upper-limit value of 8 µg/mL was used for AMB-susceptible *C. auris*. These findings suggest that Etest overestimates AMB resistance in *C. auris*, a finding that is similar to another study reported recently [29] but contrary to the results reported previously in two other studies [28,36]. An overestimation of AMB resistance in *C. auris* has also been observed with the SYO and Vitek 2-based AST methods [29,31,32]. Interestingly, an overestimation of AMB MICs by the Vitek 2 system was more pronounced for *C. auris* isolates belonging to clade I and clade IV than for the isolates belonging to clade II, clade III or clade V [32]. The EA (±1 two-fold dilution) between CLSI and SYO was 29% and the CA was only 11%, which improved to 100% when a wild-type upper-limit value of 8 µg/mL was used for AMB for the SYO method [31]. Similarly, the MICs obtained with the Vitek 2 method were also significantly higher than CLSI AMB MICs resulting in very high (69%) interpretation errors, which were reduced to only 2% major errors when a Vitek 2-specific wild-type upper limit of 16 µg/mL was used [32]. 

Previous studies have shown that several yeast species exhibiting reduced susceptibility to AMB contain genetic alterations in the *ERG* genes, particularly *ERG2, ERG3, ERG6* and *ERG11* which are involved in ergosterol biosynthesis. The mutations affecting protein function either reduce or abolish ergosterol from the fungal cell membrane and confer resistance to AMB [42,43,44,45,46]. The only molecular mechanism conferring resistance to AMB in *C. auris* fully established so far also involves genetic alterations in *ERG6* [38]. Other possible mechanisms for AMB resistance in *C. auris* include mutations in genes such as *MEC3* which is involved in DNA damage homeostasis [47]. Sanger sequencing and whole genome sequence (WGS) data for the three *C. auris* isolates scored as AMB-resistant by both the Etest and MCN-AM assay have shown genetic alterations in *ERG6*, which abolished protein function with a concomitant absence of ergosterol from the fungal cell [38]. WGS data have also been reported for 14 of 108 *C. auris* isolates scored as AMB-susceptible by both the Etest and MCN-AM assay [23]. These data showed a wild-type sequence for *ERG2, ERG3* and *ERG6* genes in all 14 isolates, as expected. The WGS data are also available for 3 of 10 *C. auris* isolates [23] which yielded discrepant results by the Etest and MCN-AM assay. All three isolates also contained a wild-type sequence for *ERG2, ERG3* and *ERG6* genes, strongly suggesting that the AMB resistance indicated by Etest in these isolates was not accurate. Taken together, these findings demonstrate how Etest likely overestimates AMB resistance in *C. auris*, although definitive clinical breakpoints are needed to confirm this observation. The MCN-AM also performed better than Vitek 2 for the AST of *C. auris* against AMB in another recent study; however, resistance mechanisms for discrepant isolates were not interrogated [48]. Taken together, our results and other recent findings strongly suggest that method-specific resistance breakpoints should be devised for an accurate AST of clinical *C. auris* isolates by rapid commercial methods for proper management of patients with invasive infections.

Our study has some limitations. (1) The AST was not performed simultaneously with the reference CLSI or EUCAST method. (2) In addition to *ERG11*, fluconazole-resistance-conferring mutations were not evaluated in other gene targets such as *TAC1B*. (3) The *ERG2, ERG3* and *ERG6* sequence data were available for only 3 of 10 isolates yielding discrepant AST results for AMB by the two methods. (4) Since this study included *C. auris* isolates belonging to South Asian clade I only, other studies should be carried out with isolates belonging to other clades. 

## 4. Materials and Methods

### 4.1. Isolation and Identification of C. auris from Clinical Specimens

A total of 121 *C. auris* isolates were used in this study (Table 3). These included 7 isolates obtained from 1 patient from Hospital A, 49 isolates obtained from 18 patients from Hospital F and 65 isolates obtained from 40 patients from Hospital G between 2016 and 2019 in Kuwait [17,33]. The clinical specimens were obtained from hospitalized patients as part of routine patient care and were processed for the culture and AST of yeast pathogens. The study was approved by the Ethics Committee of the Health Sciences Center, Kuwait University (Approval letter VDR/EC/3724). The samples were processed in BACTEC Plus blood culture bottles (Beckton Dickinson, Sparks, MD, USA) and/or on Sabouraud dextrose agar (SDA) (Difco) supplemented with chloramphenicol (50 µg/mL) plates as described previously [34,49]. The identification of all isolates as *C. auris* was indicated by the Vitek 2 yeast identification system (bioMérieux, Marcy-l’Etoile, France). The identification was also achieved by matrix-assisted laser desorption ionization time-of-flight mass spectrometry (MALDI-TOF MS) which was performed as described previously [50]. Briefly, a single colony from a fresh culture on Sabouraud dextrose agar (SDA) was used to extract surface-exposed proteins which were subjected to mass spectrometry to generate protein spectra. The identification was achieved by comparing the protein spectra with data contained in the VITEK MS database [50]. 

The identification of each isolate was confirmed by molecular methods. For this purpose, genomic DNA from each isolate was prepared by the rapid boiling method as described previously [51]. Briefly, yeast cells from a colony on an SDA plate were suspended in 1 mL of sterile water in a microcentrifuge tube containing 50 mg Chelex-100 (Sigma-Aldrich Co., St. Louis, MO, USA); the contents were heated at 95 °C for 20 min and then centrifuged. The supernatant was used as the source of genomic DNA and, typically, 2 μl was used in the various PCR assays. Species-specific identification was achieved by PCR amplification of rDNA using *C. auris*-specific CAURF and CAURR primers (Supplementary Table S1). PCR amplification was performed in 0.2 mL thin-walled PCR tubes as described previously [52]. Briefly, the reaction mixture in a final volume of 50 µl contained 1x AmpliTaq DNA polymerase buffer I, 2 units of AmpliTaq DNA polymerase, 10 pmol of CAURF and CAURR primers, 2 µL of template DNA and 100 µM of each dNTP. Cycling conditions included an initial denaturation at 95 °C for 5 min followed by 30 cycles of 95 °C for 1 min, 55 °C for 30 s, 72 °C for 1 min and a final extension at 72 °C for 10 min. PCR products (10 µL) were run on 2% (*w*/*v*) agarose gels, as described previously [53]. 

The entire internally transcribed spacer (ITS) region of rDNA was amplified using panfungal primers ITS1 and ITS4 (Supplementary Table S1) and using the PCR amplification protocol described above. PCR amplicons were purified using a PCR product purification kit (Qiagen, Hilden, Germany) according to the kit instructions. Both strands of the purified amplicons were sequenced (Sanger sequencing) using ABI Big-Dye terminator cycle sequencing kits (Thermo Fisher Scientific, Waltham, MA, USA) and internal sequencing primers ITS1FS, ITS2, ITS3 and ITS4RS (Supplementary Table S1) according to the kit instructions and as described previously [54]. Briefly, the reaction mixtures (10 μL final volume) contained 2 μL of purified amplicon, 1x reaction buffer, 2 μL of Big-Dye terminator (version 3.1) reagent and 3.2 pmol of each sequencing primer. Sequencing reaction cycling included an initial denaturation step at 95 °C for 1 min followed by 30 cycles of 1 min at 95 °C and 3 min at 60 °C. The unincorporated nucleotides and terminators were removed using a Big-Dye Xterminator kit (Thermo Fisher Scientific, Waltham, MA, USA), and the processed samples were loaded onto a DNA sequencer for electrophoresis and data collection according to the manufacturer’s instructions. The assembled sequences were compared with data available in the GenBank database using a nucleotide Basic Local Alignment Search Tool (BLASTn; https://blast.ncbi.nlm.nih.gov/BLAST/Blast.cgi?); a threshold of ≥99% sequence identity was used for species-specific identification. Clade-specific identification was achieved by 12-loci-based short tandem repeat (STR) genotyping, as described in detail previously [39].

### 4.2. In Vitro Antifungal Drug Susceptibility Testing

The *C. auris* isolates from frozen stocks were grown on SDA plates for AST and DNA extraction. The AST was performed for fluconazole and AMB using Etest strips according to the instructions supplied by the manufacturer (bioMérieux, Marcy l’Etoile, France) and as described previously [33]. For quality control purposes, parallel testing was conducted on reference strains of *Candida parapsilosis* ATCC 22019 and *Candida albicans* ATCC 90028. The AST for fluconazole and AMB was also performed using colorimetric EUCAST-based broth microdilution tests using MICRONAUT-AM AST panel for yeasts (MCN-AM) (Merlin Diagnostica GmbH, Bornheim, Germany) according to the instructions supplied by the manufacturer. Microtiter plates were incubated at 35 °C and the MIC values were read spectrophotometrically after 24 h of growth, as described previously [17]. *Candida krusei* (ATCC 6258) and *C. parapsilosis* (ATCC 22019) were also tested simultaneously for quality control. Due to a lack of *C. auris*-specific susceptibility breakpoints, tentative MIC breakpoints of ≥32 µg/mL for fluconazole and ≥2 µg/mL for AMB, as suggested by the CDC, were used for defining drug-resistant strains [55].

### 4.3. Molecular Basis of Resistance to Fluconazole and AMB

Nonsynonymous mutations Y132F or K143R mutations, conferring resistance to fluconazole in *C. auris* strains, are commonly found in the hotspot I region of the *ERG11* gene [33,36,37]. The hotspot region of the *ERG11* gene was amplified by PCR using ER11F and ERG11R primers (Supplementary Table S1) and the general amplification and cycling conditions described above. The PCR amplicons were purified using a PCR product purification kit and both strands of the purified amplicons were sequenced as described above, except that ERG11FS and ERG11RS (Supplementary Table S1) were used as sequencing primers. The assembled DNA sequences of the *ERG11* gene fragments from different isolates were analyzed for genetic alterations using sequence data from *C. auris* strain XM_018315289 as a reference (GenBank accession no. XM_018315289), as described previously [33,36]. 

The nucleotide and the encoded amino acid sequences of the *ERG6* gene, which is involved in the biosynthesis of ergosterol and is mutated in some yeast species exhibiting reduced susceptibility to AMB [42,43], were also detected for the isolates and yielded concordant results for AMB resistance. This was achieved by PCR amplification of the *ERG6* gene-coding sequence and the flanking 5′ and 3′ untranslated regions using CauERG6F and CauERG6R primers (Supplementary Table S1) and the general PCR reaction and cycling conditions described above, except that primer extension was carried out for 2 min instead of 1 min. The PCR amplicons were purified using a PCR product purification kit, and both strands of purified amplicons were sequenced as described briefly above and elsewhere [33,43], except that a forward (CauERG6FS1, CauERG6FS2 or CauERG6FS3) or reverse (CauERG6RS1, CauERG6RS2 or CauERG6RS3) sequencing primer (Supplementary Table S1) was used in the sequencing reactions. The assembled DNA sequences of the *ERG6* gene were analyzed for genetic alterations using sequence data from *C. auris* strain XM_018315289 (GenBank accession no. XM_018315289) as a reference, as described previously [33,38].

### 4.4. Statistical Analyses

The categorical variables were expressed as absolute numbers. GraphPad software (GraphPad, La Jolla, San Diego, CA, USA) was used to compare the performance of the two tests, and a Kappa coefficient (κ) value of 0–0.2, 0.21–0.4, 0.41–0.6, 0.61–0.8 and 0.81–1.0 indicated poor, fair, moderate, substantial and almost perfect agreement, respectively.

## 5. Conclusions

The EA for fluconazole MIC values from the Etest and MCN-AM assay for 121 *C. auris* isolates was only 33.1%. The CA between the two tests was 94.2% with seven discrepant results, even though all isolates contained the fluconazole resistance-conferring Y132F or K143R mutation in *ERG11.* The discrepancies were reduced to only two when an MCN-AM upper-limit value of 4 µg/mL for fluconazole-susceptible *C. auris* was used. The EA for AMB MIC values derived from the Etest and MCN-AM assay was moderate (76%), while the CA between the two tests was 91.7% (10 discrepancies). The discrepancies were completely eliminated when an Etest upper-limit value of 8 µg/mL was used for AMB-susceptible *C. auris*, supported by a lack of detectable molecular mechanisms of AMB resistance in these isolates. Our results and other recent findings strongly suggest that method-specific resistance breakpoints should be devised for accurate AST of clinical *C. auris* isolates for proper patient management.

## Figures and Tables

**Table 1:** In vitro antifungal susceptibility testing (AST) results of *C. auris* isolates (*n* = 121) against fluconazole and amphotericin B by Etest and MICRONAUT-AM (MCN-AM) assay.

Antifungal	AST	No. of Isolates with Minimum Inhibitory Concentration (MIC) (µg/mL) of																					
**Drug**	**Method**	**≤0.03**	**0.09**	**0.12**	**0.19**	**0.25**	**0.38**	**0.5**	**0.75**	**1**	**1.5**	**2**	**3**	**4**	**8**	**16**	**24**	**32**	**64**	**96**	128	192	256
Fluconazole	Etest																2	**4**	**1**	**1**	**2**	**1**	**110**
	MCN-AM													2		7		**21**	**53**		**38**		
Amphotericin B	Etest	1	1	2	6	6	12	16	18	15	31	**6**	**3**	**1**				**3**					
	MCN-AM					3		37		78		**2**		**1**									

The MIC values indicative of resistance, according to the tentative breakpoints of the CDC to antifungal drugs, are shown in bold face numbers.

**Table 2:** The minimum inhibitory concentration (MIC) range (µg/mL), modal MIC, MIC_50_, MIC_90_ and the geometric mean values of *C. auris* isolates (*n* = 121) against fluconazole and amphotericin B by Etest and MICRONAUT-AM assay.

Antifungal Drug/AST Method	MIC RANGE	Modal MIC	MIC_50_	MIC_90_	Geometric Mean ± SD
**Fluconazole**					
Etest	24-≥256	≥256	≥256	≥256	223.59 ± 54.85
MICRONAUT-AM assay	4-≥128	64	64	128	62.19 ± 39.42
**Amphotericin B**					
Etest	0.03–32	1.5	0.75	2	0.82 ± 4.89
MICRONAUT-AM assay	0.25–4	1	1	1	0.80 ± 0.41

The drug names (fluconazole and amphotericin B) are shown in bold face letters. SD, Standard deviation.

**Table 3:** Number and source of clinical *C. auris* isolates used in this study.

Specimen	No. of *C. auris*
type	Isolates *
Urine	53
Blood	29
Endotracheal secretion	15
Tracheal aspirate	5
Catheter tip	4
Wound/pus swab	4
Bronchoalveolar lavage	2
Oral cavity swab	2
Tissue biopsy	2
Sputum	1
Pleural fluid	1
Eye swab	1
Axilla swab	1
Vaginal swab	1
**Total**	**121**

* Some patients yielded more than 1 isolate from the same or different specimens.

## Data Availability

All relevant data are available in the manuscript.

## Supplementary Materials

The following supporting information can be downloaded at: https://www.mdpi.com/article/10.3390/antibiotics13090840/s1, Supplementary Table S1. Nucleotide sequences and specific purpose of primers used in PCR amplification or DNA sequencing of various genomic regions of *C. auris* isolates and the expected sizes of amplicons, where applicable, in base pairs (bp) [33,34,54].
