# Supplementary material for: Evaluation of Etest and MICRONAUT-AM Assay for Antifungal Susceptibility Testing of Candida auris: Underestimation of Fluconazole Resistance by MICRONAUT-AM and Overestimation of Amphotericin B Resistance by Etest
Source: Antibiotics (Basel). 2024 Sep 4;13(9):840. doi: 10.3390/antibiotics13090840 (PMC11428412; doi:10.3390/antibiotics13090840)
Supplement: Supplementary file 1 [file antibiotics-13-00840-s001.zip › antibiotics-3130295-supplementary.pdf]

S1 Table. Nucleotide sequences and specific purpose of primers used in PCR-amplification or DNA sequencing of various genomic regions of *C. auris* isolates and the expected sizes of amplicons, where applicable, in base pairs (bp)

Primer name	Nucleotide sequence	Direction	Purpose	Amplicon Size* (bp)	Reference
CAURF	5′-ATTTTGCATACACACTGATTTG-3′	Forward	*C. auris*-specific PCR amplification of rDNA	276	34
CAURR	5′-CGTGCAAGCTGTAATTTTGTGA-3′	Reverse	*C. auris*-specific PCR amplification of rDNA		34
ITS1	5′-TCCGTAGGTGAACCTGCGG-3′	Forward	Panfungal PCR amplification of rDNA	~400-900	54
ITS4	5′-TCTTTTCCTCCGCTTATTGATATGC-3′	Reverse	Panfungal PCR amplification of rDNA		54
ITS1FS	5′- ACCTGCGGAAGGATCATT-3′	Forward	Panfungal DNA sequencing primer for rDNA	N. A.	54
ITS3	5′-TCGCATCGATGAAGAACGCAGC-3′	Forward	Panfungal DNA sequencing primer for rDNA	N. A.	54
ITS4RS	5′- GATATGCTTAAGTTCAGCG-3′	Reverse	Panfungal DNA sequencing primer for rDNA	N. A.	54
ITS2	5′-TCGCTGCGTTCTTCATCGATGC-3′	Reverse	Panfungal DNA sequencing primer for rDNA	N. A.	54
ERG11F	5′-GTGGGCTCTGCTGTTGTTTA-3′	Forward	*C. auris*-specific PCR amplification of *ERG11*	330	33
ERG11R	5′-CAAAACTTCCTCTTGGATTCTG	Reverse	*C. auris*-specific PCR amplification of *ERG11*		33
ERG11FS	5′-GCTCTGCTGTTGTTTACGGA-3′	Forward	Sequencing primer for *C. auris ERG11* fragment	N. A.	33
ERG11RS	5′-ACTTCCTCTTGGATTCTGGGCA-3′	Reverse	Sequencing primer for *C. auris ERG11* fragment	N. A.	33
CauERG6F	5′-GCATGCGCGTTTATATAAACGCA-3′	Forward	*C. auris*-specific PCR amplification of *ERG6*	1338	This study
CauERG6R	5′-TACGACTCGACATTTCGTCGA-3′	Reverse	*C. auris*-specific PCR amplification of *ERG6*		This study
CauERG6FS1	5′-GCGCGTTTATATAAACGCACCA-3′	Forward	Sequencing primer for *C. auris ERG6* gene	N. A.	This study
CauERG6FS2	5′-GCACTATTTGGCCTACAAGA-3′	Forward	Sequencing primer for *C. auris ERG6* gene	N. A.	This study
CauERG6FS3	5′-GTACAAGAGGGACGTTGCCGA-3′	Forward	Sequencing primer for *C. auris ERG6* gene	N. A.	This study
CauERG6RS1	5′-ACTCGACATTTCGTCGATGTG-3′	Reverse	Sequencing primer for *C. auris ERG6* gene	N. A.	This study
CauERG6RS2	5′-ATCTCAAAGCCAACGTTCTTCA-3′	Reverse	Sequencing primer for *C. auris ERG6* gene	N. A.	This study
CauERG6RS3	5′-GCCAACATCCAAAACCTTCATG-3′	Reverse	Sequencing primer for *C. auris ERG6* gene	N. A.	This study

*Amplicon sizes (in base pairs, bp) are based on various combinations of forward primers with their reverse primer. The amplicon sizes for rDNA with panfungal primers ITS1 and ITS4 vary among different *Candida* species due to variations in the length of internal transcribed spacer-1 and internal transcribed spacer-2 regions. N. A., not applicable
